# Exercise Induced Rhabdomyolysis with Compartment Syndrome and Renal Failure

**DOI:** 10.1155/2014/735820

**Published:** 2014-07-06

**Authors:** Mary Colleen Bhalla, Ryan Dick-Perez

**Affiliations:** Summa Akron City Hospital, 525 East Market Street, P.O. Box 2090, Akron, OH 44304-1619, USA

## Abstract

Exertional rhabdomyolysis is sequela that is occasionally seen after strenuous exercise. The progression to compartment syndrome or renal failure is a rare complication that requires prompt recognition and treatment to prevent morbidity (Giannoglou et al. 2007). We present a case of a 22-year-old college football player who presented to the emergency department (ED) after a typical leg workout as part of his weight conditioning. He was found to have rhabdomyolysis with evidence of renal insufficiency. His condition progressed to bilateral compartment syndrome and renal failure requiring dialysis. After bilateral fasciotomies were performed he had resolution of his compartment syndrome. He continued to be dialysis dependent and had no return of his renal function at discharge 12 days after admission.

## 1. Introduction

Rhabdomyolysis is the result of muscular injury through either physical forces or nonphysical means such as exertion. The initial cellular injury leads to swelling of the cells and release of cellular contents including electrolytes, myoglobin, and creatinine kinase, which is the basis of diagnosis [1, 2]. Creatinine kinase (CK) levels typically peak in 1–3 days and then begin to decrease [2]. However in rare cases the rhabdomyolysis is extensive enough to lead to compartment syndrome and worsening muscular injury secondary to impaired blood flow [3]. The release of myoglobin and uric acid can lead to renal injury and in some cases renal failure [2, 4]. The treatment for rhabdomyolysis is a renal protective strategy of hydration and diuresis as well as preventing further muscle injury [2, 3]. To accomplish this, compartments must be examined, as compartment syndrome requires emergent surgical treatment to prevent permanent muscle damage as well as further elevation in myoglobin, electrolytes, and uric acid resulting in worsening renal injury and potential cardiac dysrhythmias [1, 5].

## 2. Case Presentation

### 2.1. History

A 22-year-old college African American football player presented to the ED 2 days after a workout with his football team. According to the patient the workout took place at the school's indoor workout facility and was a typical off-season workout. Through the course of the workout he did strenuous weight lifting exercises targeting the lower body including a “series of squats” that he did not further explain.

He had been seen in an urgent care center the day before for bilateral thigh pain and a sore back. He was given a prescription for oxycodone/acetaminophen 5 mg/325 mg two tabs every six hours as well as an unknown muscle relaxant and sent home to continue symptomatic management. He had worsening pain and dark colored urine so he presented to the emergency department for reevaluation. At presentation he was complaining of bilateral thigh pain as well as lower back pain that was worse with ambulation and dark colored urine. He had no history or exercise induced muscle damage and denied the use of performance enhancing substance or steroids and had no history of alcohol, tobacco, or drug use. He also denied fevers, chill, nausea, or vomiting. He was otherwise healthy and took no prescription or over-the-counter medications on a regular basis.

### 2.2. Physical

Vital signs were as follows: blood pressure 154/98 mmHg, heart rate 97 beats/min, and respiratory rate 16 breaths/min with 100% pulse oximetry on room air. He was a well-nourished and well-developed male in no acute distress. Head, eyes, ears, nose, and throat exam was noncontributory. Heart was at regular rate and rhythm with no murmurs noted and lungs clear to auscultation bilateral. Abdomen was soft nontender and nondistended. His lower extremities were noted to be soft and nontender with intact sensation. Upon admission it was noted that he had developed tenderness in his bilateral thighs but maintained full range of motion and 2+ dorsalis pedis and posterior tibial pulses with intact sensation.

### 2.3. Diagnostics and Clinical Course

Initial chemistry panel was significant for a potassium 6.0 mmol/L (3.5–5.1 mmol/L), blood urea nitrogen (BUN) 31 mg/dL (7–25 mg/dL), creatinine (Cr) 4.7 mg/dL (0.60–1.5 mg/dL), glomerular filtration rate (GFR) of 4.74 mL/min (>60 mL/min), uric acid 12.6 mg/dL (3.5–7.2 mg/dL), ALT 989 Unit/L (0–56 Units/L), AST 6303 Unit/L (0–31 Unit/L), and CK > 200,000 Unit/L (39–297 Unit/L). A complete blood count showed a normal white blood cell count with a hemoglobin of 17.7 g/dL (14–17.5 g/dL) and a hematocrit of 53.2% (42–50%). His urine showed myoglobin 191 ng/mL (<50 ng/mL). He was treated initially with a two-liter normal saline bolus. After he was found to have elevated potassium he was treated with sodium polystyrene sulfonate and calcium gluconate for his elevated potassium. He was placed on maintenance fluids at 250 cc/hour and admitted to the intensive care unit. Given his elevated liver enzymes, he had a hepatitis panel, viral panel, and anabolic steroid panel that were all negative.

Since he was complaining of worsening leg pain and had laboratory evidence or muscle damage there was a concern for compartment syndrome and orthopedic surgery was consulted. Using a Stryker Intracompartmental Pressure Monitor System compartment pressures were measured and he was found to have anterolateral compartment syndrome with pressures of 64 mmHg on the right and 44 mmHg on the left (critical pressure > 30 mmHg). In the evening of admission he continued to have worsening pain and swelling so he was taken to the operating room (OR) for emergent fasciotomies of bilateral anterolateral thigh compartments. At time of surgery he was noted to only have 30 degrees of knee flexion and opening of the fascia causing a large release of pressure and protuberance of muscle through the skin incision. Muscle appeared healthy with no evidence of necrosis. Negative-pressure wound therapy devices were placed and he returned to the OR to have the incision closed at a later date.

The patient was started on dialysis on hospital day two and remained on dialysis throughout his course. His labs normalized during his hospital stay and he was discharged on hospital day 12 for continued outpatient physical therapy and dialysis. At discharge he continued to show significant renal impairment with a GFR of 5.0 mL/min (>60 mL/min); nephrology felt that his renal function would eventually return to normal. Labs performed three months later showed a BUN 21 mg/dL (7–25 mg/dL), a Cr 0.93 mg/dL (0.60–1.5 mg/dL), and a GFR > 60 mL/min (>60 mL/min) which were all in the normal range.

## 3. Discussion

Physical exertion is a potential cause of rhabdomyolysis and progression to renal failure secondary to myoglobinuria is seen in 10%–50% of patients [2]. A rare consequence of rhabdomyolysis is progression to acute compartment syndrome. This can cause further elevations in CK, which leads to severe renal damage, electrolyte abnormalities, and permanent muscle damage [6]. There are multiple risk factors that have been identified and there are steps that can be taken by athletes to prevent exertional rhabdomyolysis.

The risk factors that most pertain to athletes are temperature elevations, hypokalemia, and the use of nutritional supplements [7, 8]. Education may prevent the occurrence; however, when exertional rhabdomyolysis does present to the ED clinicians need to keep high index of suspicion for compartment syndrome especially when symptoms worsen [6, 9].

The patient we presented had no known risk factors for exertional rhabdomyolysis. We do not have any laboratory evaluation on the patient prior to his episode of exertional rhabdomyolysis to determine if he had a history of hypokalemia that he was unaware of. However the exercise environment was climate controlled and there was no sudden increase in physical activity as this was a typical workout for the patients. One area that was not addressed during his admission was his hydration status prior to and during the workout, as dehydration can predispose a person to exertional muscle damage. The patient was African American; however red blood cell morphology showed no evidence of sickle cell anemia.

The classic 5 Ps, pain, pallor, pulselessness, paresthesias, and paralysis, of compartment syndrome are rarely seen and only pain is consistently present in review articles, though still unreliable [5, 9, 10]. Since the physical exam is a poor screen for compartment syndrome if there is a concern for compartment syndrome in the ED compartment pressures should be checked to allow for rapid diagnosis and treatment [6].
